# Effects of adenosine receptor overexpression and silencing in neurons and glial cells on lifespan, fitness, and sleep of *Drosophila melanogaster*

**DOI:** 10.1007/s00221-023-06649-y

**Published:** 2023-06-19

**Authors:** Debarati Bhattacharya, Jolanta Górska-Andrzejak, Terence Al L. Abaquita, Elżbieta Pyza

**Affiliations:** Department of Cell Biology and Imaging, Jagellonian University, Kraków, Poland

**Keywords:** Adenosine, Circadian rhythms, Photoreceptors, Synaptic plasticity, BRP protein

## Abstract

A single adenosine receptor gene (*dAdoR)* has been detected in *Drosophila melanogaster*. However, its function in different cell types of the nervous system is mostly unknown. Therefore, we overexpressed or silenced the *dAdoR* gene in eye photoreceptors, all neurons, or glial cells and examined the fitness of flies, the amount and daily pattern of sleep, and the influence of *dAdoR* silencing on Bruchpilot (BRP) presynaptic protein. Furthermore, we examined the *dAdoR* and *brp* gene expression in young and old flies. We found that a higher level of dAdoR in the retina photoreceptors, all neurons, and glial cells negatively influenced the survival rate and lifespan of male and female *Drosophila* in a cell-dependent manner and to a different extent depending on the age of the flies. In old flies, expression of both *dAdoR* and *brp* was higher than in young ones. An excess of dAdoR in neurons improved climbing in older individuals. It also influenced sleep by lengthening nighttime sleep and *siesta*. In turn, silencing of *dAdoR* decreased the lifespan of flies, although it increased the survival rate of young flies. It hindered the climbing of older males and females, but did not change sleep. Silencing also affected the daily pattern of BRP abundance, especially when *dAdoR* expression was decreased in glial cells. The obtained results indicate the role of adenosine and dAdoR in the regulation of fitness in flies that is based on communication between neurons and glial cells, and the effect of glial cells on synapses.

**Supplementary Information:**

The online version contains supplementary material available at 10.1007/s00221-023-06649-y.

## Introduction

Adenosine receptors are G protein-coupled receptors (GPCRs) that have been detected in vertebrates and invertebrates (Fredholm et al. 2011). In mammals, four different subtypes of AdoR have already been identified: A1, A2A, A2B, and A3. They are crucial in the functioning of the cardiovascular and immune systems (Linden 2001; Mayne et al. 2001; Ohta and Sitkovsky 2001), as well as in the peripheral and central nervous systems (Sebastio and Ribiero 2000). The action of AdoRs appears to be particularly important in the brain, since their ligand, adenosine, is the main molecule that regulates brain activity (Boison 2007). Adenosine is produced by ATP breakdown and is released from glia and neurons (Latini and Pedata 2001) to affect synaptic transmission and neuronal membrane properties by activating inhibitory A1 or excitatory A2A receptors (Chen et al. 2013). It also controls and facilitates the release of synaptic mediators (Sebastio and Ribiero 2000).

Adenosine is also known to regulate sleep (Haulica et al. 1973). It is a natural sleep-promoting substance that accumulates during prolonged waking (Benington et al. 1995) and after sleep deprivation (Huston et al. 1996; Porkka-Heiskanen et al. 1997, 2000). It has been shown to promote slow wave sleep by inhibiting arousal centers comprising the mesopontine tegmentum, hypothalamus (Liu et al. 2008), and basal forebrain (Alam et al. 1999; Arrigoni et al. 2001; Morairty et al. 2004; Rainnie et al. 1994; Thakkar et al. 2003).

In invertebrates, studies on AdoR are scarce. In *Drosophila*, a single *dAdoR* gene (CG9753) was described (Dolezelova et al. 2007). Its sequence revealed a 70% sequence similarity to *Anopheles gambiae* (392aa) and *Apis mellifera* (462aa) and a 38% similarity in the N-terminal region to human A2AR (Dolezelova et al. 2007). The level of expression of *dAdoR* in different cell lines and tissues of *Drosophila* shows a notable variation. However, the highest expression has been reported in the nervous system (Dolezelova et al. 2007; Kucerova et al. 2012). In larvae, *dAdoR* is expressed in the optic lobe of the brain, the ring gland, the imaginal discs, and the salivary glands (Dolezelova et al. 2007; Kucerova et al. 2012). It is GPCR that activates adenylate cyclase (Kucerova et al. 2012), leading to cAMP production and calcium signaling (as the A2B receptor in mammals). Insect AdoR activation is essential to facilitate energy metabolism during development (Dolezelova et al. 2007). The overexpression of *dAdoR* in all tissues has been found to be lethal for larvae and pupae, while tissue-specific expression results in milder lethal phenotypes (Dolezelova et al. 2007). Interestingly, a specific diet, for example a 10% addition of sucrose to the rearing medium, can prevent this lethality (Buch et al. 2008).

In *Drosophila*, *dAdoR* signaling plays a role in synaptic plasticity, stress response, immune protection, hematopoiesis, and intestinal stem cell activity (Knight et al. 2010; Bajgar et al. 2015; Mondal et al. 2011; Poernbacher and Vincent 2018; Xu et al. 2020). The A2A adenosine receptor is known to mediate many anti-inflammatory properties of adenosine (Linden 2001; Mayne et al. 2001; Ohta and Sitkovsky 2001), increasing the rate of wound healing (Cronstein 2006; Montesinos et al. 2000, 2003, 2007, 2015).

The fruit fly is a perfect model to study mechanisms of sleep (Ly et al. 2018), which can be easily measured in this species. The fruit fly has decreased sensory responsiveness during sleep and shows a rebound after sleep deprivation (Hendricks et al. 2000; Nitz et al. 2002; Tononi 2000; Van Swinderen et al. 2004). After the discovery of *dAdoR* in *Drosophila*, adenosine could be further studied for its role in the regulation of sleep and rest/activity.

Sleep is important and helps to improve fitness (Potdar et al. 2018), learning and memory (Diesel et al. 2015), energy conservation (Stahl et al. 2017), immune functions (Besedovsky et al. 2012), and waste clearance (Van Alphen et al. 2021). Sleep in *Drosophila* is regulated by neurotransmitters and stimulants similar to those of mammals and characterized as total sleep, daytime sleep, and nighttime sleep. Total sleep refers to sleep that occurs during the light and dark periods (Liu et al. 2015). Daytime sleep, also known as nap or *siesta*, occurs in the middle of the day and helps to protect flies against high temperature. The *siesta* is sexually dimorphic since males show more consolidated and longer daytime sleep than females (Shaw et al. 2000; Koh et al. 2008). On the other hand, nighttime sleep is characterized by a higher arousal threshold (Faville et al. 2015; Van Alphen et al. 2013).

In addition to the circadian rhythm in sleep and activity, synaptic plasticity in the brain also shows a circadian oscillation. It has especially been studied in the visual system of flies (Pyza 2002). In the first optic neuropil (lamina), most synapses are the tetrad synapses formed between the photoreceptor terminals and four lamina post-synaptic cells. The *Drosophila* synapse is characterized by a presynaptic element called a T bar, and its main scaffolding protein is Bruchpilot (BRP). BRP is homologous to the mammalian ELKS/CAST family of synaptic proteins (Wagh et al. 2006) that facilitates vesicle release (Kittel et al. 2006). The T bar is composed of two BRP isoforms, 190 kD (BRP-190) and 170 kD (BRP-170) (Wagh et al. 2006). Both isoforms are recognized by the NC82 anti-BRP monoclonal antibody NC82 (Matkovic et al. 2013).

This protein is known to exhibit circadian plasticity. In WT flies, the level of this protein oscillates twice during the day, at the beginning of the day and at the beginning of the night (Woznicka et al. 2015; Górska-Andrzejak et al. 2013). This oscillation resembles the bimodal activity pattern seen in *Drosophila* with morning and evening peaks of activity (Wheeler et al. 1993; Górska-Andrzejak et al. 2013). The morning peak of BRP is under the control of light, whereas the evening peak is controlled by the circadian clock (Górska-Andrzejak et al. 2013). During the day, the expression of BRP depends on the CRY protein (Damulewicz et al. 2020) that is a circadian photoreceptor (Emery et al. 1998) and regulator of synaptic plasticity in the visual system (Damulewicz and Pyza 2011; Damulewicz et al. 2017).

The present study aimed to examine the role of *dAdoR* overexpression or silencing in three cell types: photoreceptors, neurons, and glial cells in survival, fitness, night sleep, *siesta* (daytime sleep), and the presynaptic protein BRP abundance during the day. In addition, we examined mRNA level of *dAdoR* and *brp* in young and old flies.

## Materials and methods

### Animals

For experiments, we used Canton-S wild-type flies and the following transgenic strains: *GMR*-*Gal4* (*GMR-Gal4w*^*1118*^*; P{GMR-Gal4.w-}2/CyO*, received from the laboratory of Dr. Ralf Stanewsky), *elav*-*Gal4* (*P{Gal4-elav.L}2/CyO*, from Bloomington *Drosophila* Stock Center, No 8765), *repo*-*Gal4*4 (*w*^*1118*^*; P{Gal4) repo-/TM3,Sb*^*1*^, from Bloomington *Drosophila* Stock Center, No 7415;), *UAS-AdoR* (received from the laboratory of Dr Eva Dolezelova), *UAS-VALIUM10* (*P {UAS-GFP.VALIUM10} attP2*; from Bloomington *Drosophila* Stock Center, No 35786), and *UAS-AdoR-*^*RNAi*^ (No 1386 from Vienna *Drosophila* Resource Centre; VDRC). Table 1 lists the transgenic lines of *Drosophila* with references.

**Table 1:** *Drosophila* transgenic lines with references

Fly lines	References
*GMR-Gal4*	Li et al. (2012)
*elav-Gal4*	Ogienko et al. (2020)
*repo-Gal4*	Halter et al. (1995)
*UAS-AdoR*	Dolezelova et al. (2007)
*UAS-AdoR*^RNAi^	Xu et al. (2020)
*UAS-VALIUM 10*	Ni et al. (2009), Wagner et al. (2021)

We targeted retina photoreceptors, all neurons, and glial cells using GMR-*Gal4,* pan-neuronal *elav-Gal4,* and pan-glial *repo*-*Gal4* drivers, respectively. These driver strains were crossed with the *UAS*-*AdoR* strain (*GMR/elav/repo*-*Gal4* > *UAS*-*AdoR*) to overexpress *AdoR* and with Canton-S flies (*GMR/elav/repo*-*Gal4* > *CS*) to obtain sibling controls verifying the genetic background. For silencing studies, the driver strains were crossed with the *UAS*-*AdoR*^RNAi^ strain (*GMR/elav/repo*-*Gal4* > *UAS*-*AdoR*^RNAi^) and with the strain *UAS*-*VALIUM* (*GMR/elav/repo*-*Gal4* > *UAS*-*VALIUM*) to obtain individuals with silenced expression of *AdoR* and a relevant control group of individuals, respectively.

Experiments were carried out on males and females of the first generation (F1) of these crosses. The flies were fed a standard yeast, cornmeal, and agar diet. They were kept in a light/dark cycle (LD12:12, 12 h of light and 12 h of darkness), at a constant temperature of 25 °C.

### Survival assay

In the survival assay, 1-day-old males and females were placed in vials (30 flies per vial) with cornmeal medium. Dead individuals were counted every day until the end of the experiment. To maintain optimal rearing conditions, the flies were transferred on fresh food every two days. It was done without anesthetizing the flies with CO_2_, which could affect their activity. The survival rate for each group is presented as a Kaplan–Meier survival curve in which the percentage of live flies is plotted against their age. The median survival (MS) of each group designates the day in which 50% of the flies were dead.

### Climbing assay

We used a geotactic climbing assay based on the natural behavior of *Drosophila* to climb against gravity (negative geotaxis) to test general locomotion and fitness of flies. For the assay, 30 males and 30 females were loaded into empty vials of 70 ml volume (φ = 3.5 cm). Each vial had a line marked 5 cm above the bottom. During the test, the vials were vigorously tapped to force the flies to descend to the bottom, to record the number of flies that climbed above the line in 15s.

The climbing abilities of 7-, 14-, 30-, and 60-day-old flies were assessed at each time in three trials. The test was always carried out at the beginning of the day (at ZT1—1 h after the beginning of the light phase in LD12:12), when flies are typically very active. The experiment was carried out in a dark room to avoid increased motor activity in response to light stimuli or other distractions.

### Locomotor activity and sleep analysis

Males of 1- to 2-day-old were used for this experiment. They were placed in small tubes (φ = 5 mm) to record their locomotor activity and rest cycles in monitors of the Drosophila Activity Monitoring System (DAMS) (TriKinetics, Waltham, MA, USA; Nall et al. 2016). Each monitor housed 32 tubes/flies. The tubes contained the agar–sugar medium (5% sugar and 2% agar) that is commonly used in DAMS (Nall et al. 2016) at one end and the plug at the other end. The system recorded interruptions of the infrared beam emitted by the monitors that were caused by flies walking inside the tubes. The activity of the flies was recorded for 13 days: 7 days in LD12:12 and 6 days in constant darkness (DD). During recording, the monitors were kept in incubators with set temperature, humidity, and light conditions (Rosato and Kyriacou 2006). Data were obtained in 1 min bins. Since *Drosophila* sleep is identified as a period of minimum 5 min of inactivity (Huber et al. 2004), for sleep examination, each hour the 5-min bins of immobility (sleep) were examined. Sleep and activity analysis was performed using ShinyR-DAMS online software (Cichewicz and Hirsh 2018). The sleep phenotypes of the flies were determined based on the second day of activity recording in LD 12:12.

### Immunofluorescent labeling of the Bruchpilot (BRP) presynaptic protein

7- to 10-day-old males were immobilized with CO_2_ and decapitated in a drop of fixative: 4% formaldehyde (PFA) in 0.1 M phosphate buffer (PB). The flies were decapitated on specific ZTs—“*Zeitgebers*” (time givers), 4 times during the day: at the beginning of the day (ZT1) and the night (ZT13), in the middle of the day (ZT4) and the night (ZT16) (in LD 12:12, ZT0 denotes the end of the night/beginning of the day and ZT12 denotes the end of the day/beginning of the night). For each time point (ZT), 30 flies were sacrificed. Decapitation during the dark part of the cycle, at ZT13 and ZT16, was conducted in dim red light, using a dissecting microscope equipped with red exit filters on fiber optic light guides (Pyza and Meinertzhagen 1996).

After tissue fixation and cryoprotecting infiltration in a 25% sucrose solution, head cryo-sections were cut and incubated with mouse Mab nc82 [Developmental Studies Hybridoma Bank (DSHB), IA], which recognizes the C-terminus of the BRP protein (Kittel et al. 2006; Wagh et al. 2006). After several washes in 0.01 M sodium phosphate buffer (PBS) containing 0.02% Triton-X (Sigma), sections were incubated with goat anti-mouse Cy3 conjugated secondary antibodies (Jackson ImmunoResearch Laboratories). The preparations were examined using a Zeiss LSM 780 Meta confocal microscope after a final wash and mounting in Vectashield medium (Vector). Images of the first optic neuropil (lamina) from different ZTs, showing cartridges in the longitudinal section, were collected (7–10 images/individuals per ZT) using identical image acquisition parameters.

### Immunolabeling quantification

The fluorescence intensity of the BRP-specific immunolabeling (brightness) in the distal and proximal parts of 5–10 lamina cartridges per individual was evaluated using ImageJ software (NIH, Bethesda) as the Mean Grey Value that corresponds to the sum of the gray values of all pixels in a selected area divided by the number of pixels within the selection (ImageJ divides the range of gray values between Min and Max in 16-bit images into 256 bins). The Mean Grey Value for each image/individual was used for statistical analysis of differences that occurred between *AdoR*^*RNA*i^ flies and their controls at different time points.

### RNA isolation, cDNA synthesis, and quantitative PCR

Male and female flies, 7- or 30-day-old, were decapitated at ZT1. Heads were fixed in 100% ethanol for 2 h, and brains were isolated. Approximately 25 brains were used for a sample, and each experiment was repeated five times.

Total RNA isolation was performed using TriReagent (MRC Inc., Irvine, CA, USA) according to the manufacturer’s protocol. The RNA quality and quantity were assessed using Nanodrop 2000 (Thermo Fisher Scientific, MA, USA). cDNA was synthesized using a High-Capacity cDNA Reverse Transcription Kit (Thermo Fisher Scientific, Vilnus, Lithuania) with random primers according to the provider’s instruction. The analysis of the expression levels for the target genes listed in Table 2 was done using StepOnePlus Real-Time PCR System and SYBR Green Master Mix (KAPA Biosystems, Cape Town, South Africa) in the presence of specific primer sequences. Product specificity was controlled by Primer-BLAST and gel electrophoresis and further assessed by melting curve analysis. A standard curve was used to calculate gene expression levels. The number of target gene copies was normalized to the geometric mean of the *rpl32* gene, the housekeeping gene.

**Table 2:** Primer sequences of the genes used in the study

Gene		Sequence	Accession number
*rpl32*	F:	5′-CATGGACTACCCAGAAACC-3′	CG7939
	R:	5′-CAGTATCGATCCACGGACAC-3′	
*AdoR*	F:	5′-TATGCTAAGCTGTCGCACAAATG-3′	CG9753
	R:	5′-AGCACGTGTATAAAAAGTGCCA-3′	
*brp*	F:	5′-GAGAAGGCCACCTGCGAAAT-3′	CG42344
	R:	5′-GTTGTGAGTACCTGCGAACG-3′	

### Statistics

All statistical analyses were performed with GraphPad Prism 5.0 software (Graph Pad, San Diego, CA, USA) and the R/R Studio freeware statistical package version 4.2.0 (http://www.R-project.org/, accessed 16 March 2023). The percentage of survival between genotypes was analyzed using the log-rank test (Mantle–Cox). Mean differences in climbing, sleep, BRP level, and gene expression level were compared using the Mann–Whitney *U* test (differences between two groups) or the nonparametric counterpart of ANOVA, the Kruskal–Wallis test (one-way test) followed by *Dunn’s* or *Conover–Iman’s* test for multiple comparisons of independent samples. In each analysis, probability values of *p* < 0.05 were established for significant differences. The error bars represent standard deviations (SD).

## Results

### Increased dAdoR in photoreceptors, neurons, and glial cells affects flies’ survival

**Fig. 1:**
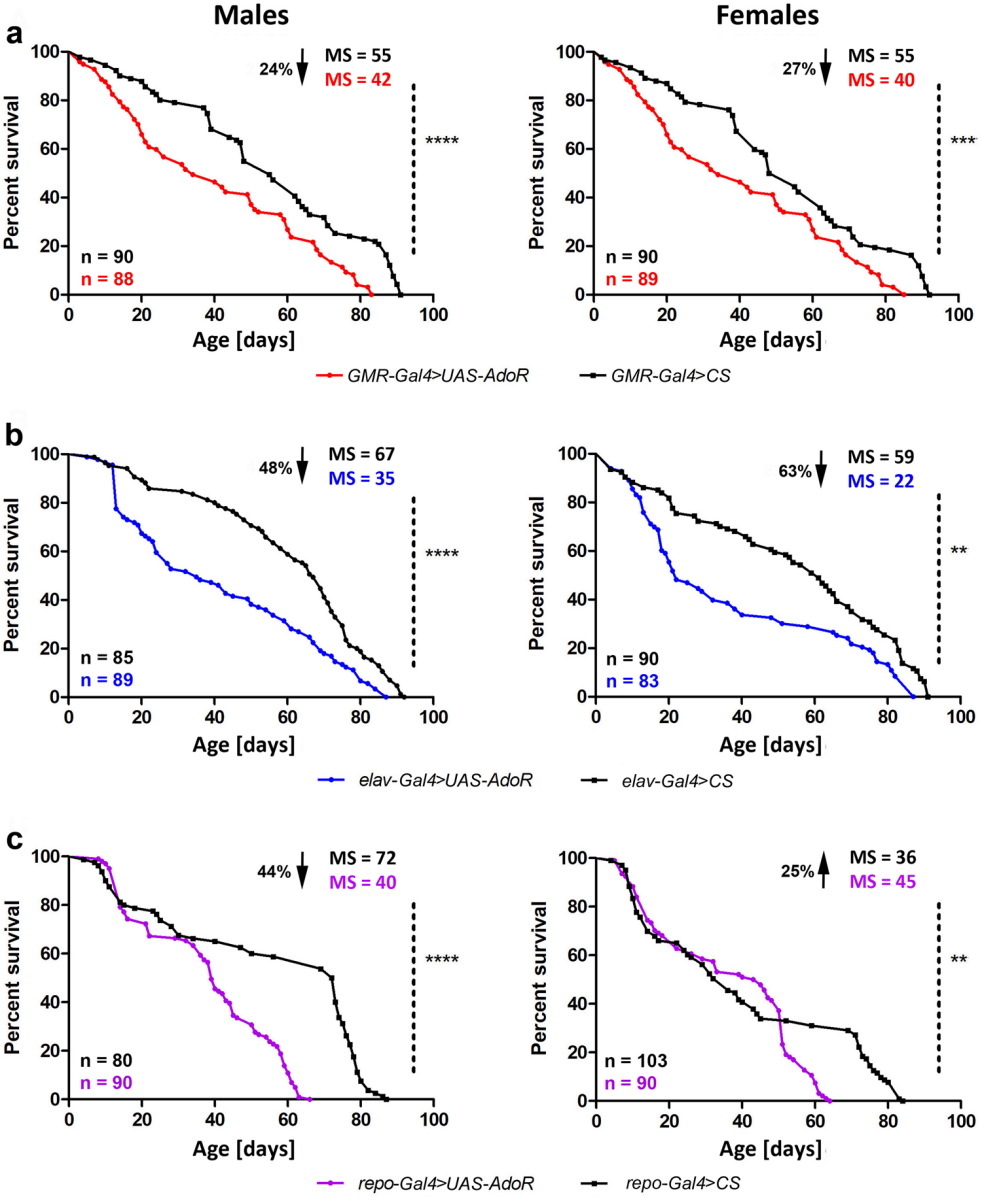
Kaplan–Meier survival curves for males and females with overexpressed *dAdoR* in photoreceptors (red), neurons (blue), and glial cells (purple), and for the relevant control groups (black). Survival differences were checked using the log-rank test (Table 3). Statistically significant differences between curves are indicated with asterisks; *p < 0.01, **p < 0.001, ****p < 0.0001. MS median survival. The arrow accompanied by the percentage indicates the value of MS decrease or increase

The higher level of dAdoR in photoreceptors, neurons, and glial cells negatively influenced flies’ survival as revealed by the Kaplan–Meier survival curves of males and females with overexpressed *dAdoR* in photoreceptors, all neurons, or glial cells (Fig. 1, Table 3).

**Table 3:** Long-rank test statistics for survival analysis of males (gray lines) and females (white lines) with *AdoR* overexpression/silencing in photoreceptors (*GMR*-), neurons (*elav*-), and glial cells (*repo*-) compared to control

Groups compared	Overexpression Log-rank test Statistic	Overexpression Log-rank test *p* value	Silencing Log-rank test Statistic	Silencing Log-rank test *p* value
***GMR***-*Gal4* > *UAS*-*AdoR* vs. GMR-*Gal4* > *CS*	16.4	< 0.0001		
*GMR*-*Gal*4 > *UAS*-*AdoR* ^RNAi^ vs. *GMR*-*Gal4* > *UAS-VALIUM*			5.0	< 0.05
*GMR*-*Gal4* > *UAS*-*AdoR* vs. *GMR*-*Gal4* > *CS*	11.4	< 0.001		
*GMR*-*Gal4* > *UAS*-*AdoR* ^RNAi^ vs. *GMR*-*Gal4* > *UAS-VALIUM*			10.1	< 0.01
*elav*-*Gal4* > *UAS*-*AdoR* vs. *elav*-*Gal4* > *CS*	18.5	< 0.0001		
*elav*-*Gal4* > *UAS*-*AdoR* ^RNAi^ vs. *elav*-*Gal4* > *UAS-VALIUM*			7.7	< 0.01
*elav*-*Gal4* > *UAS*-*AdoR* vs. *elav*-*Gal4* > *CS*	9.6	< 0.01		
*elav*-*Gal4* > *UAS*-*AdoR* ^RNAi^ vs. *elav*-*Gal4* > *UAS-VALIUM*			6.7	< 0.01
*repo*-*Gal4* > *UAS*-*AdoR* vs. *repo*-*Gal4* > *CS*	47.6	< 0.0001		
*repo*-*Gal4* > UAS-*AdoR* ^RNAi^ vs. *repo*-*Gal4* > *UAS-VALIUM*			10.7	< 0.01
*repo*-*Gal4* > *UAS*-*AdoR* vs. *repo*-*Gal4* > *CS*	7.1	< 0.01		
*repo*-*Gal4* > *UAS*-*AdoR* ^RNAi^ vs. *repo*-*Gal4* > *UAS-VALIUM*			5.2	< 0.05
Males				
Females				

Overproduction of *dAdoR* mRNA in photoreceptors and other cells expressing *GMR*, decreased median survival (MS) and the lifespan of both males and females (Fig. 1a). *GMR-Gal4* is generally used as a driver for the eye. However, *GMR-Gal4* has a broad expression profile (Li et al. 2012).

Half of the males and females of *GMR*-*Gal4* > *UAS-AdoR* lived 42 and 40 days, respectively, while the males and females of the control groups lived 55 days. A similar but stronger trend was observed when *dAdoR* overexpression was induced in all neurons (Fig. 1b). The MS for *elav*-*Gal4* > *UAS-AdoR* flies was even more reduced. It was 35 and 22 days for males and females, respectively. Overexpression of *dAdoR* in photoreceptors and in all neurons decreased the survival of 1- to 50-day-old flies rather than the older ones. The maximum individual lifespan for both *GMR*-*Gal4* > *UAS-AdoR* and *elav*-*Gal4* > *UAS-AdoR* flies was 80 days, which was 10 days shorter than the maximum lifespan of the control flies.

Interestingly, the influence of *dAdoR* overexpression on flies’ survival was quite different when overexpression was induced in glial cells. Here, the main effect, a rapid decrease in survival compared to control flies, was observed in older flies that lived more than 40 days (Fig. 1c). Consequently, the maximum individual lifespan was reduced to 66 and 64 days for males and females, respectively. In control flies, the maximum individual lifespan was 84 days for males and 89 days for females.

### The decrease in dAdoRs in photoreceptors, neurons, and glial cells mildly affects survival of flies

**Fig. 2:**
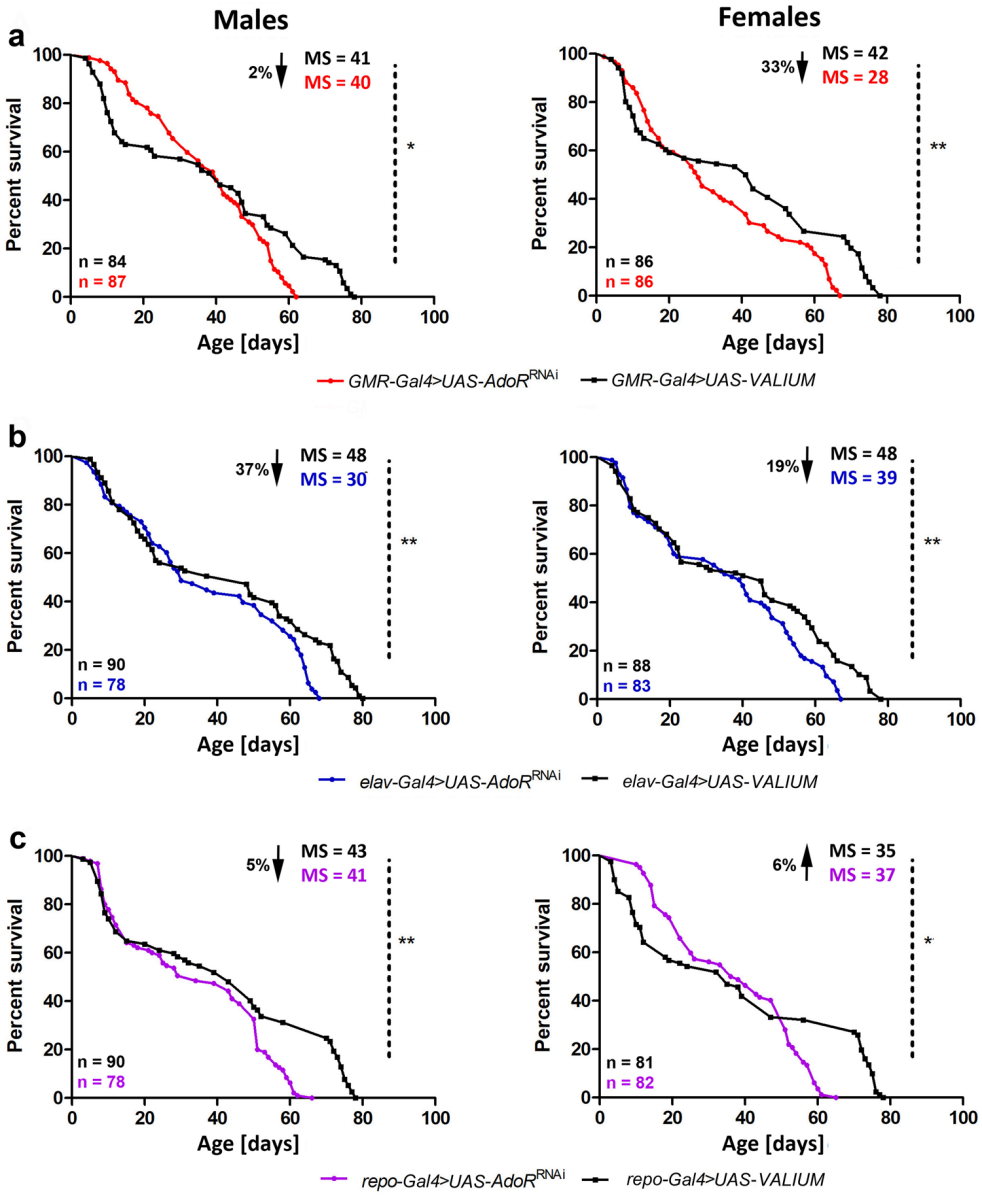
Kaplan–Meier survival curves for males and females with silenced expression of *dAdoR* in photoreceptors (red), neurons (blue) and glial cells (purple), and for the relevant control groups (black). Survival differences were checked using the log-rank test (Table 3). Statistically significant differences between curves are indicated with asterisks; *p < 0.01, **p < 0.001, ****p < 0.0001. MS median survival. The arrow accompanied by the percentage indicates the value of MS decrease or increase

The Kaplan–Meier survival curves of males and females with silenced expression of *dAdoR* in photoreceptors, all neurons, and glial cells also differed from the curves of control flies (Fig. 2, Table 3), although the differences were smaller than those caused by the overexpression of *dAdoR* (Fig. 1).

The median survival of *GMR*-*Gal4* > *UAS*-*AdoR*^RNAi^ males (with silenced *dAdoR* in photoreceptors) and males in the control group was 40 and 41 days, respectively. However, the survival of males with silenced *dAdoR* in photoreceptors was better than that of males in the control group before their 40th day of life and worse later with aging (Fig. 2a). *GMR*-*Gal4* > *UAS*-*AdoR*^RNAi^ males survived up to 62 days compared to 78 days in the control. In the case of female survival, the initially positive effect of *dAdoR* silencing in photoreceptors was observed only until they were 20-day-old. The survival of older females (with silenced *AdoR* in photoreceptors) was worse than that of the control group. They lived up to 67 days compared to 78 days in the control. The MS of *GMR*-*Gal4* > *UAS*-*AdoR*^RNAi^ and control females was 28 and 42 days, respectively.

A similar trend was observed in *elav*-*Gal4* > *UAS*-*AdoR*^RNAi^ flies with decreased *dAdoR* mRNA in neurons. However, neither in males nor in females was there a positive effect of neuronal silencing of *dAdoR* in the first phase of the experiment. The MS of flies with silenced *dAdoR* in neurons was 30 days for males (vs. 48 days for the control) and 39 days for females (vs. 45 days for the control) (Fig. 2b), while the maximum lifespan of flies with silenced *dAdoR* in neurons was 68 days for males (vs. 80 days for the control) and 67 days for females (vs. 78 days for the control).

Silencing *dAdoR* in glial cells decreased MS in males (4.7%) and increased (5.7%) in females (Fig. 2c). In the latter, the survival rate was better than in control flies in the first stage of the experiment. The maximum lifespan of *repo*-*Gal4* > *UAS*-*AdoR*^RNAi^ males was 68 days (vs. 78 days for the control) and the maximum lifespan of *repo*-*Gal4* > *UAS*-*AdoR*^RNAi^ females was 65 days (vs. 78 days for the control).

### dAdoRs affect the fitness of flies when expressed in neurons and glia

**Fig. 3:**
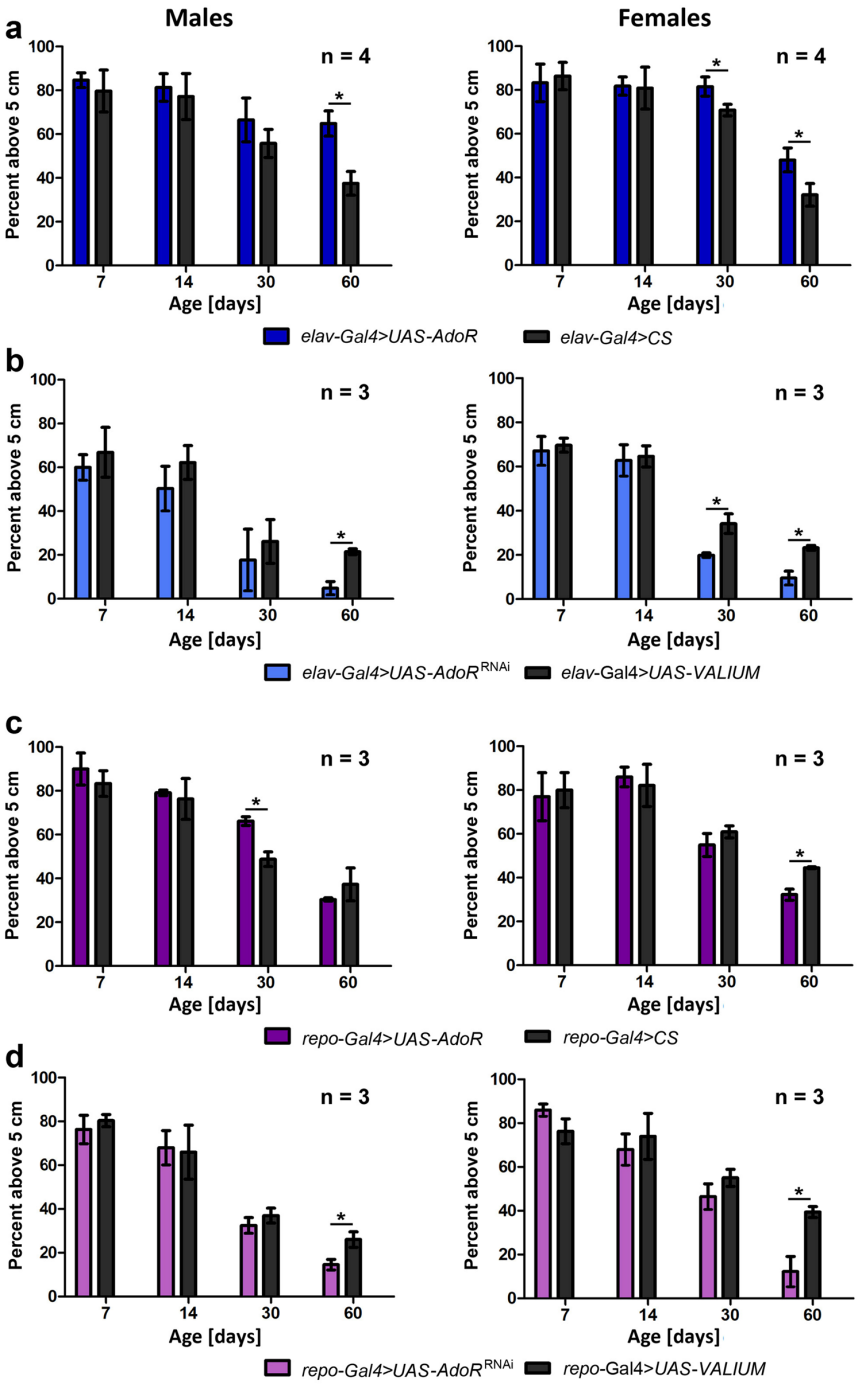
Climbing of males and females with *dAdoR* overexpression and silencing in neurons (**a**, **b**) and glial cells (**c**, **d**). There were 30 males or 30 females in the vial in which the test was performed. The percentage of flies that climbed above 5 cm is plotted over the age of the flies. Bars show the mean of 3–4 repeats. Some statistically significant differences between experimental and control flies are indicated with asterisks; *p < 0.01, **p < 0.001, ****p < 0.0001, others are included in Table 4

To test the locomotion and fitness of flies with overexpressed or silenced *dAdoR*, the climbing behavior of flies was evaluated in the negative geotactic climbing assay (Fig. 3). Typically, the climbing ability of flies decreases with age (Rhodenizer et al. 2008), and this was also observed in both the experimental and control groups in our experiment (Fig. 3, Table 4). There were significant differences between the 7-day-old and 14-day-old flies and 60-day-old flies (Table 4).

**Table 4:** Results of the Kruskal–Wallis test (K–W test) and the *post hoc Conover–Iman’s* test (C–I test; *p* < 0.05 in all cases) for the climbing assay of 7-, 14-, 30- and 60-day-old males (gray lines) and females (white lines) of different experimental and control groups

Groups	K-W test Statistic	K-W test *p* value	C-I test	
*elav*-*Gal4* > *UAS*-*AdoR*	10.6	< 0.05	7, 14 vs 60	
*elav*-*Gal4* > *CS*	12.7	< 0.01	7, 14 vs 30, 60	
*elav*-*Gal4* > *UAS*-*AdoR*	8.8	< 0.05	7, 14 vs 60	
*elav*-*Gal4* > *CS*	11.2	< 0.05	7, 14 vs 60	
*elav*-*Gal4* > *UAS*-*AdoR* ^RNAi^	9.6	< 0.05	7 vs 30, 60; 14 vs 60	
*elav*-*Gal4* > *UAS-VALIUM*	8.8	< 0.05	7 vs 30, 60; 14 vs 60	
*elav*-*Gal4* > *UAS*-*AdoR* ^RNAi^	9.3	< 0.05	7 vs 30, 60; 14 vs 60	
*elav*-*Gal4* > *UAS-VALIUM*	9.7	< 0.05	7 vs 30, 60; 14 vs 60	
*repo*-*Gal4* > *UAS*-*AdoR*	10.4	< 0.05	all combinations	
*repo*-*Gal4* > *CS*	9.5	< 0.05	7 vs 30, 60; 14 vs 60	
*repo*-*Gal4* > *UAS*-*AdoR*	9.7	< 0.05	7 vs 60; 14 vs 30, 60	
*repo*-*Gal4* > *CS*	9.4	< 0.05	7 vs 30, 60; 14 vs 60	
*repo*-*Gal4* > UAS-*AdoR* ^RNAi^	9.97	< 0.05	7 vs 30, 60; 14 vs 60	
*repo*-*Gal4* > *UAS-VALIUM*	10.4	< 0.05	all combinations	
*repo*-*Gal4* > UAS-*AdoR* ^RNAi^	10.4	< 0.05	all combinations	
*repo*-*Gal4* > *UAS-VALIUM*	9.3	< 0.05	7 vs 30, 60; 14 vs 60	
Males				
Females				

However, the climbing of old flies with overexpressed or silenced expression of *dAdoR* differed significantly from the climbing of control flies of the same age.

The climbing ability of 60-day-old males with *dAdoR* overexpression in neurons was significantly better than control flies of the same age (Mann–Whitney *U*-test, *U* = 0.0, *p* < 0.05), just as climbing of 60- and 30-day-old females with *dAdoR* overexpression was better than 60- and 30-day-old females of the control group (Mann–Whitney *U*-test, *U* = 0.0, *p* < 0.05: Fig. 3a). In turn, silencing of *dAdoR* in neurons (Fig. 3b) had the opposite effect on climbing. It significantly decreased the climbing ability of 60-day-old males (Mann–Whitney *U*-test, *U* = 0.0, *p* < 0.05) as well as 60- and 30-day-old females (Mann–Whitney *U*-test, *U* = 0.0, *p* < 0.05) in comparison to their control groups. Thus, the effects of both overexpression and silencing were stronger in females than in males (and therefore became more significant earlier, in 30-day-old females).*dAdoR* overexpression in glial cells (Fig. 3c) showed that 30-day-old males had significantly better climbing abilities than control males (Mann–Whitney *U*-test, *U* = 0.0, *p* < 0.05). However, this could not be further visible in older, 60-day-old males. The climbing ability of 60-day-old females with *dAdoR* overexpression in the glia was considerably worse than those of control flies of the same age group (Mann–Whitney *U*-test, *U* = 0.0, *p* < 0.05; Fig. 3c). Interestingly, silencing of *dAdoR* in glial cells caused a further decline in climbing abilities in 60-day-old males and females (Mann–Whitney *U*-test, *U* = 0.0, *p* < 0.05; Fig. 3d).

Changes in adenosine receptor abundance only in eye photoreceptors did not cause significant differences in the climbing ability of flies.

### Adenosine receptor signaling in photoreceptors, neurons, and glial cells affects *Drosophila* sleep

**Fig. 4:**
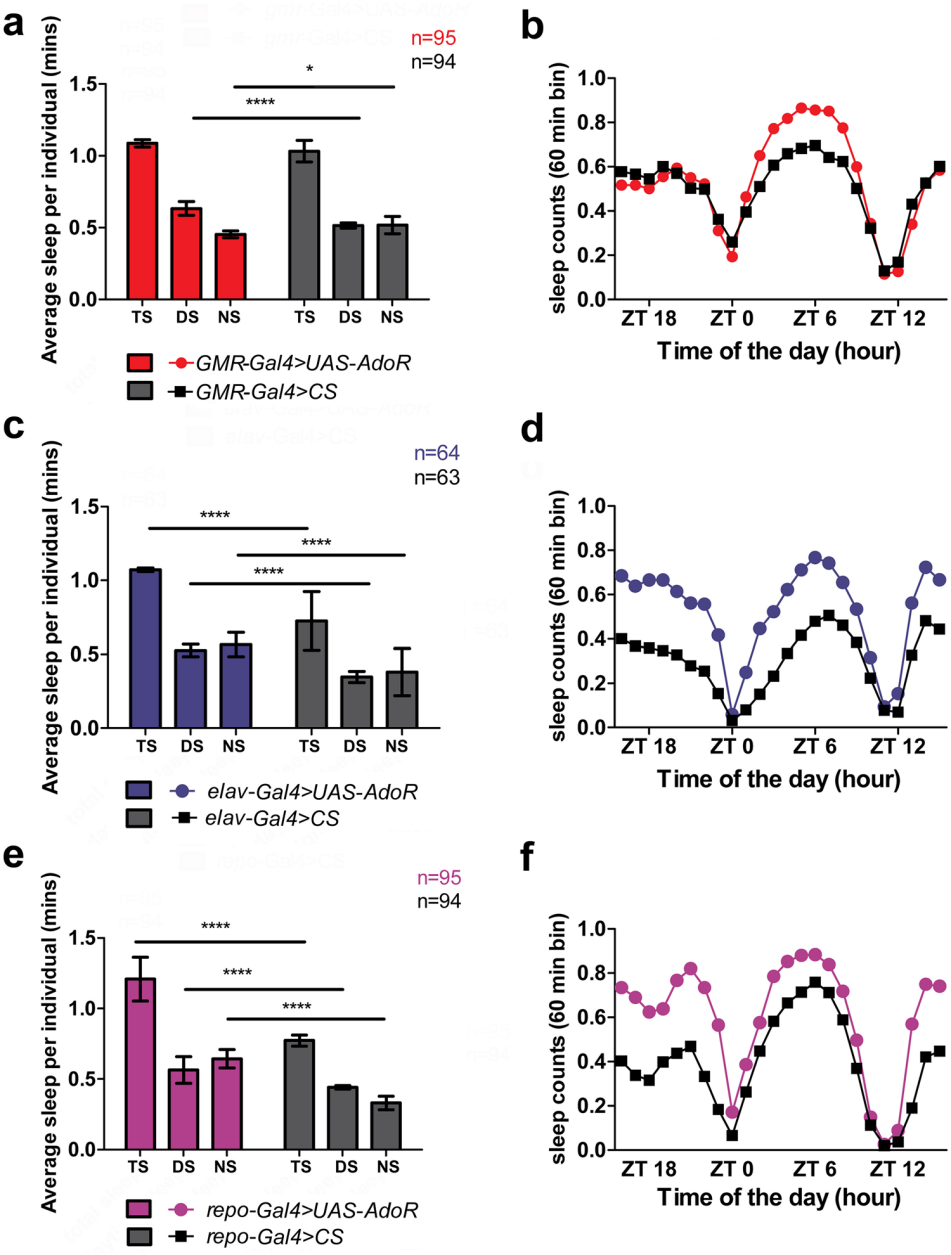
Average (± SD) total sleep (TS), daytime sleep (DS), and nighttime sleep (NS) (**a**, **c**, **e**), as well as daily average sleep profiles showing the number of sleep counts in 60-min bins (**b**, **d**, **f**) on the second day of activity recording in LD12:12. Data show sleep of males with *dAdoR* overexpression in photoreceptors (red), neurons (blue), and glial cells (magenta). They were obtained in two or three repetitions, and the number of individuals (**a**, **c**, **e**) or repetitions (**b**, **d**, **f**) is shown in the figure. Statistically significant differences are indicated with asterisks: *p < 0.05, ****p < 0.0001

The obtained results indicate that adenosine receptor signaling in photoreceptors, neurons, and glial cells influences day (*siesta*) and night sleep in *Drosophila* (Fig. 4) when intensified due to receptor overexpression.

When *dAdoR* was overexpressed in photoreceptors, the total sleep of flies (*GMR*-*Gal4* > *UAS-AdoR*) did not change; however, the daytime sleep of flies was significantly longer (Mann–Whitney *U-*test, *U* = 3020, *p* = 0.0001), while their nighttime sleep was slightly shorter (Mann–Whitney *U-*test, *U* = 3675, *p* = 0.05) than the daytime and nighttime sleep of *GMR*-*Gal4* > *CS* control flies, respectively (Fig. 4a). There were increasingly more sleep counts in the average sleep profile of the experimental flies from ZT1 to ZT5, 6, and 7. Later during the day, the number of sleep counts decreased, but until ZT9, it was still larger than in control flies (Fig. 4b). Therefore, the most significant differences between the experimental flies and their sibling controls occurred during the light part of the cycle, when the photoreceptors are active (Fig. 4b).

When *dAdoR* was overexpressed in all neurons, the flies (*elav*-*Gal4* > *UAS-AdoR*) slept significantly more than the control flies during the light and dark parts of the 24 h cycle (Fig. 4c). Their total sleep was significantly longer (Mann–Whitney *U-*test, *U* = 892, *p* = 0.0001) due to the 35% increase in daytime sleep (Mann–Whitney *U-*test, *U* = 1032, *p* < 0.0001) and the 36% increase in nighttime sleep (Mann–Whitney *U-*test, *U* = 1031, *p* < 0.0001). There were also more sleep counts in the average sleep profile of the experimental flies than in the profile of their siblings during both day (ZT0–ZT12) and night (ZT12–ZT0) (Fig. 4d).

Overexpression of *dAdoR* in glial cells (Fig. 4e), as in neurons, induced a significant increase in both daytime (Mann–Whitney *U* test, *U* = 2408, *p* < 0.05) and nighttime sleep (increase of 48%; Mann–Whitney *U-*test, *U* = 1254, *p* < 0.05) of *repo*-*Gal4* > *UAS-AdoR* flies). Consequently, total sleep of flies increased by 35%; Mann–Whitney *U-*test, *U* = 1230, *p* < 0.05). However, in this case, daytime sleep increased only by 21%, whereas nighttime sleep increased by 48%. There were also clearly more sleep counts at all time points of the night (ZT13–ZT23) in the average sleep profile of the experimental flies than in their sibling controls (Fig. 4f).

In parallel, our results showed a decrease in night locomotor activity in flies with *dAdoR* overexpression. We also observed that most flies with overexpression or silencing of *dAdoR* were rhythmic in locomotor activity and period of the rhythm was similar in experimental and control flies (data not shown).

It indicates that the circadian clock was not substantially distorted by the overexpression of *dAdoR* in any of the cell types, as the period of the free-running rhythm of locomotor activity of flies in constant darkness (DD) was similar to that of the control individuals.

**Fig. 5:**
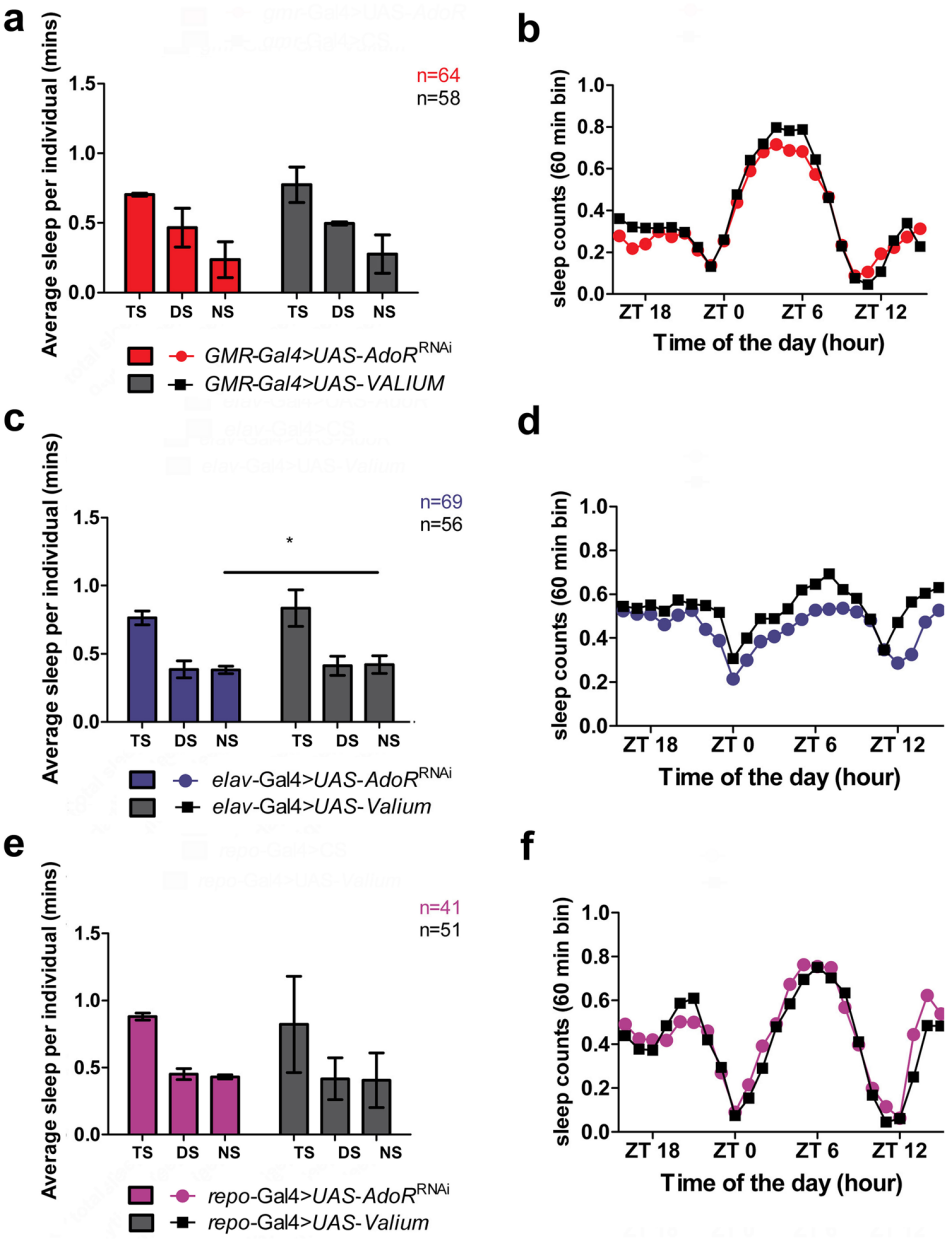
Average (± SD) total sleep (TS), daytime sleep (DS), and nighttime sleep (NS) (**a**, **c**, **e**), as well as daily average sleep profiles showing the number of sleep counts in 60-min bins (**b**, **d**, **f**) on the second day of activity recording in LD12:12. Data show sleep of males with silenced expression of *dAdoR* in photoreceptors (red), neurons (blue), and glial cells (magenta). They were obtained in two or three repetitions, and the number of individuals (**a**, **c**, **e**) or repetitions (**b**, **d**, **f**) is shown in the figure. Statistically significant differences are indicated with asterisks: *p < 0.05

The genetic cross of driver lines (*Gal4*) with carriers of the *UAS- AdoR* construct produced F1 flies that revealed a clear increase in the amount of sleep compared to control siblings and in a cell-specific fashion (Fig. 4). However, when the same driver lines were crossed with the *UAS*- *AdoR*^RNAi^ carriers, F1 flies showed almost no sleep differences from the control flies. Silencing of *dAdoR* in photoreceptors, neurons, or glial cells did not cause significant changes in the amount of total, daytime and nighttime sleep (Fig. 5a, e), except for the small decrease in nighttime sleep that was observed in *elav*-*Gal4* > *UAS*- *AdoR*^RNAi^ flies (Fig. 5c). There was also no change in the length of the circadian period of locomotor activity rhythm (data not shown). When silencing the *dAdoR* gene was in clock neurons, there was no effect on sleep of the fruit fly (data not shown). This lack of effect may be due to wake signaling after down-regulation of dAdoR in the small number of neurons.

### *AdoR* silencing influences the daily pattern of BRP abundance

**Fig. 6:**
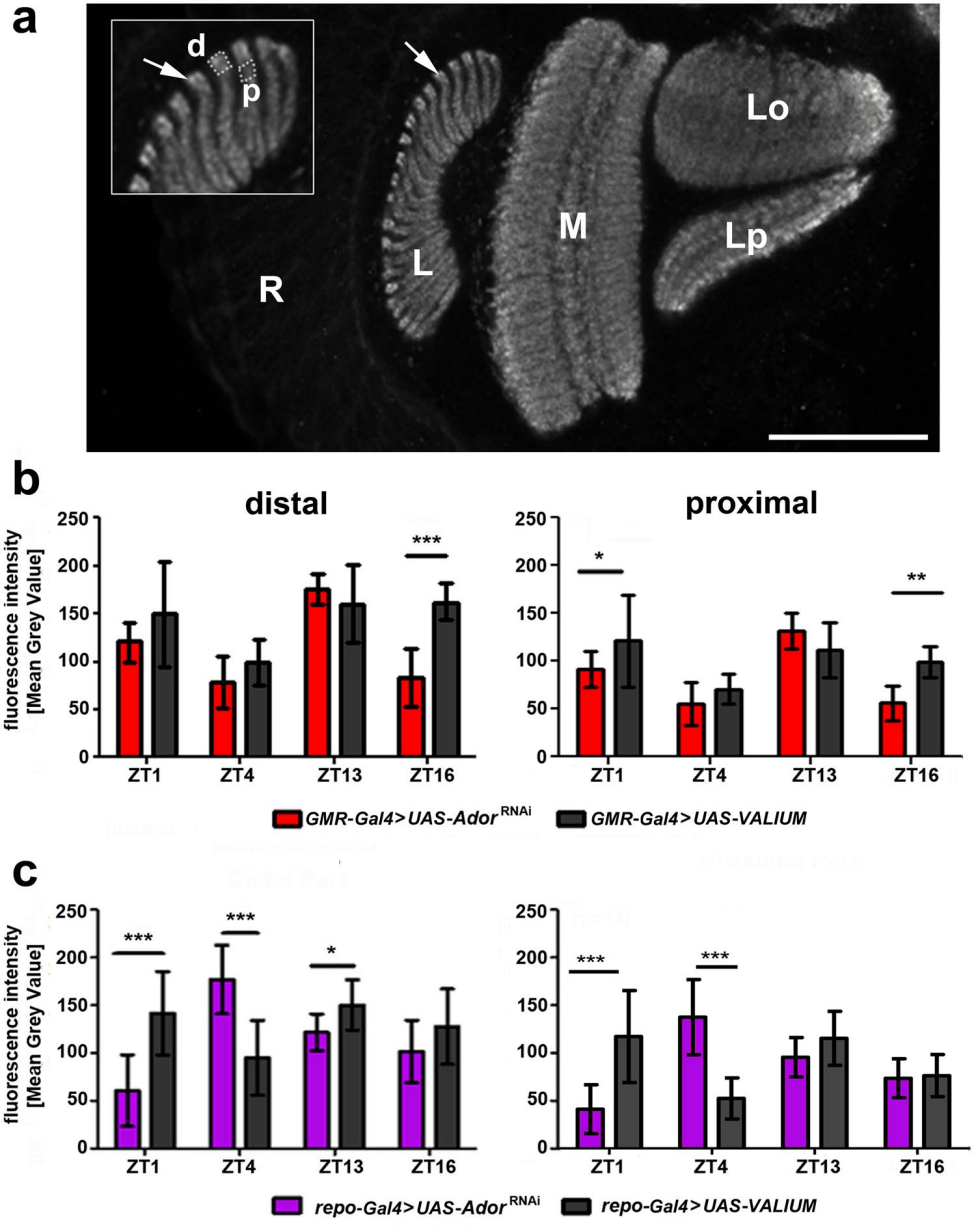
BRP abundance in the first optic lobe (lamina) of *Drosophil*a as revealed by immunostaining with Mab nc82. The level of BRP was measured in the lamina (L), in the distal (d) and proximal (p) depths (dotted areas in the insert) of its synaptic columns or cartridges (arrows) of control flies and flies with *dAdoR* silencing in photoreceptors (**b**, red) and glial cells (**c**, magenta). The intensity of BRP-related fluorescence that corresponds to BRP abundance is represented as the Mean Grey Value (*n*_ZT1,4,13,16_ = 10–11). R retina, L lamina, M the second optic neuropil or medulla, Lo and Lp the third optic neuropil or lobula, which consists of lobula (Lo) and lobula plate (Lp); Scale bar: 50 µm. Statistically significant differences are indicated with asterisks; *p < 0.05, ***p < 0.001

Since our studies showed the decrease in *Drosophila* fitness after silencing of *dAdoR* in neurons or glial cells, we decided to check the influence of silencing on the functioning of *Drosophila* synapses and examined the abundance of the presynaptic protein, Bruchpilot (BRP). We chose to monitor the level of BRP in synaptic units (cartridges) of the first visual neuropil or lamina (Fig. 6a), where it shows daily and circadian fluctuations (Górska-Andrzejak et al. 2013). We decided to examine its level at four time points (in ZT1, ZT4, ZT13, and ZT16) and in flies with silencing of *dAdoR* in the lamina photoreceptors, the terminals of which are the main neuronal components of the lamina cartridges, as well as in glial cells, which enwrap each cartridge.

### The influence of *dAdoR* silencing in photoreceptors

Our results showed that the level of BRP-related fluorescence in the distal and proximal parts of the lamina cartridges (Fig. 6a) changed significantly during the day in control (distal part: K–W test, *H* = 13.76, p < 0.001; proximal part: K–W test, *H* = 13.88, *p* < 0.001) and experimental flies with silenced expression of *AdoR* in photoreceptors (distal part: K–W test, *H* = 27.34, *p* < 0.0001; proximal part: K–W test, *H* = 28.01, p < 0.0001).

In the distal lamina of control flies (Fig. 6b, left), the lowest level of fluorescence was observed in the middle of the day, at ZT4. There were significant differences between ZT4 and ZT1 (*p* < 0.05), ZT13 (*p* < 0.01), and ZT16 (*p* < 0.05). The experimental group with silenced *AdoR* in the photoreceptors showed low fluorescence not only at ZT4, but also at ZT16. There were significant differences between ZT13, when fluorescence was the highest, and ZT4 (*p* < 0.001), and ZT16 (*p* < 0.01). Therefore, the pattern of daily changes in BRP varied in the distal lamina of control and experimental flies. The highest (49%) and statistically significant difference between them occurred in the middle of the night, at ZT16 (49%; Mann–Whitney *U-*test,* U* = 5, *p* < 0.001; Fig. 6b, left).

At the proximal depth of the lamina (Fig. 6b, right), ZT-dependent changes in the level of BRP-related fluorescence between the control and experimental flies were very similar to those in the distal lamina. In control flies, the lowest level of fluorescence was measured at ZT4 (it was significantly lower than at ZT1 and ZT13, for both *p* < 0.01), whereas in the experimental flies, it was low at ZT4 and at ZT16 (in both cases, it was significantly lower than at ZT13, *p* < 0.001). However, significant differences between control and experimental flies occurred not only in ZT16 (44%; Mann–Whitney *U-*test,* U* = 5, *p* < 0.001) but also in ZT1 (24.5%; Mann–Whitney *U-*test,* U* = 26, *p* < 0.05).

### The influence of *dAdoR* silencing in glial cells

The level of BRP-related fluorescence in the distal and proximal depths of the lamina cartridges (Fig. 6c) also changed significantly during the day in experimental flies with silenced expression of *AdoR* in glial cells (distal part: K–W test, *H* = 24.04, *p* < 0.0001; proximal part: K–W test, *H* = 23.88, *p* < 0.0001). Their controls also revealed significant changes in the fluorescence intensity during the day (distal part: K–W test, *H* = 65, *p* < 0.05; proximal part: K–W test, *H* = 17.51, *p* < 0.001).

In the distal lamina of control flies (Fig. 6c, left), like in the controls for *AdoR* silencing in photoreceptors (Fig. 6b, left), the lowest fluorescence was observed in ZT4 (but the statistically significant difference occurred only between ZT4 and ZT13; *p* < 0.05). In turn, the experimental group with silenced *AdoR* in glial cells showed the lowest fluorescence at ZT1. There was a significant difference between ZT1 and ZT4 (*p* < 0.001), when the fluorescence intensity was the highest. In the experimental group, fluorescence was also significantly higher at ZT4 than at ZT16 (*p* < 0.01). Consequently, the pattern of daily changes in BRP of the distal lamina in control and experimental flies with silenced *AdoR* in glial cells varied greatly. Significant changes occurred during the day – at ZT1 (57%; Mann–Whitney *U-*test,* U* = 7, *p* < 0.001) and ZT4 (46%; Mann–Whitney *U-*test,* U* = 7, *p* < 0.001), and at the beginning of the night – at ZT13 (19%; Mann–Whitney *U-*test,* U* = 20, *p* < 0.05).

In the proximal lamina (Fig. 6c, right), ZT-dependent changes in the level of BRP-related fluorescence in control and experimental flies were very similar to those in the distal lamina. The lowest fluorescence intensity was observed at ZT4. It was significantly lower than at ZT1 (*p* < 0.01) and at ZT13 (p < 0.01). The experimental group with silenced *AdoR* in glial cells again showed the lowest fluorescence at ZT1 and the highest at ZT4. There were statistically significant differences between ZT1 and ZT4 (*p* < 0.001), as well as between ZT1 and ZT13 (*p* < 0.05). The fluorescence intensity at ZT4 was also significantly higher than at ZT16 (*p* < 0.05). In the proximal lamina, significant changes between control and experimental flies with silenced *AdoR* in the glia occurred only during the day—at ZT1 (65%; Mann–Whitney *U-*test,* U* = 6, *p* < 0.001) and ZT4 (62%; Mann–Whitney *U-*test,* U* = 6, *p* < 0.001) (Fig. 6c, right).

### The level of *AdoR* and *brp* mRNA changes with age

**Fig. 7:**
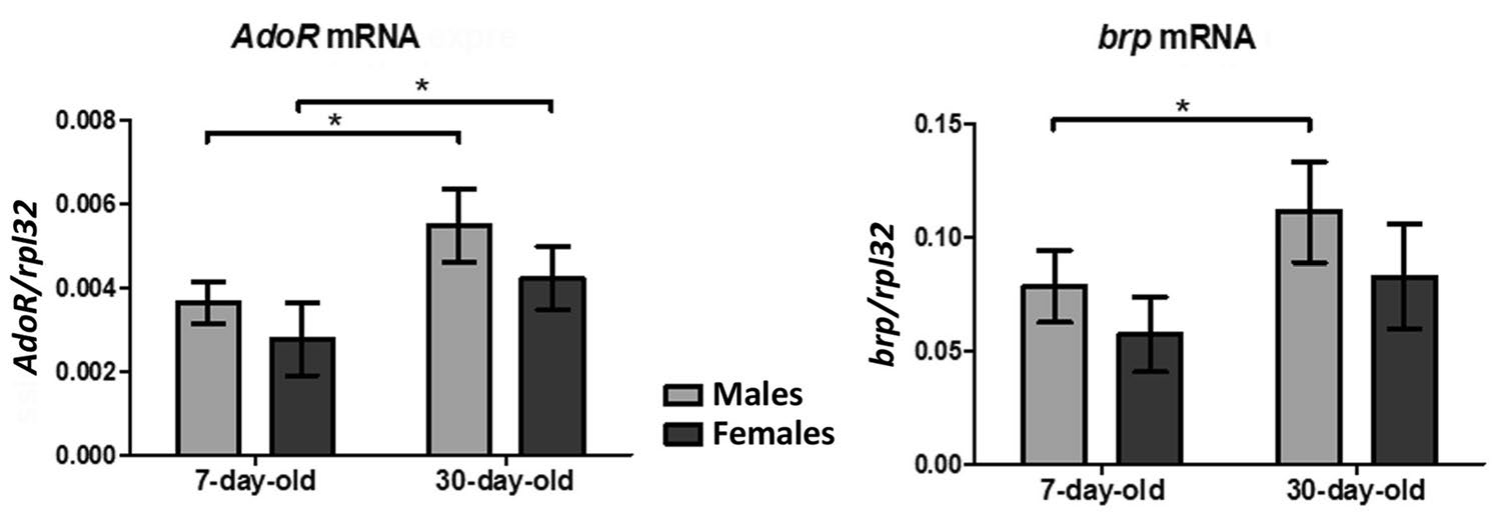
Relative quantities of the expression of *dAdoR* and *brp* mRNA (mean ± SD) comparing to the house keeping gene *rpl32* in the brain of 7-day and 30-day-old Canton-S flies kept in LD12:12. Flies were collected for analysis at ZT1. Approximately 25 brains were used for a sample and 5 samples for each group were analyzed (*n* = 5). Statistically significant differences are indicated with an asterisk; *p < 0.05

To find the background for the observed behavioral changes, we checked whether the level of *AdoR* and *brp* mRNA changes with age in wild-type Canton-S *Drosophila* (Fig. 7). Our results showed that the level of *AdoR* mRNA is higher in 30-day-old than in 7-day-old flies (males: Mann–Whitney *U-*test,* U* = 0.0, *p* < 0.05; females: Mann–Whitney *U-*test,* U* = 2.0, *p* < 0.05). However, it was similar in males and females of a given age group (7-day-old flies: Mann–Whitney *U-*test,* U* = 5, *p* > 0.05; 30-day-old flies: Mann–Whitney *U-*test,* U* = 2.0, *p* > 0.05).

The level of *brp* mRNA changed significantly with age only in males. It was higher in 30-day-old males than in 7-day-old males (Mann–Whitney *U-*test,* U* = 2, *p* < 0.05). In females there was a similar trend, but the difference between 30- and 7-day-old females was not statistically significant (Mann–Whitney *U-*test,* U* = 3.0, *p* > 0.05). There were also no significant differences between either 7-day-old (Mann–Whitney *U-*test,* U* = 4.0, *p* > 0.05) or 30-day old (Mann–Whitney *U-*test,* U* = 4.0, *p* > 0.05) males and females.

## Discussion

In the present study, we report novel functions of dAdoR in *Drosophila*. We found that adenosine and AdoR are involved in the regulation of fitness in flies, their longevity and behavior that depend on the interactions between neurons and glial cells, especially the effect of AdoR in glial cells on synapses. In addition, we observed that *dAdoR* gene expression changes with age since the level of mRNA is higher in older flies, probably due to the decrease in the level of the dAdoR protein with aging. The same trend we observed in BRP and *brp* mRNA levels. The older flies had less BRP than the young ones and a higher level of *brp* mRNA. This seems to be a common mechanism in aging, when the protein level decreases, the expression of the genes encoding them increases (Gonskikh and Polacek 2017). Our studies clearly showed that a low level of AdoR in old flies results in low fitness and increased sleep during the day and night.

Although a decrease or increase in dAdoR resulted in shortening of longevity, the survival rate that reveals the general strength and vigor of flies showed that increased adenosine signaling is harmful to young flies, as it leads to increased mortality in the initial stage of life of adult flies. This finding is consistent with another study showing that an excess of extracellular adenosine causes the death of *Drosophila* larvae and pupae (Dolezal et al. 2005). However, we also observed an increase in survival in middle-aged individuals with overexpressed *dAdoR*. Therefore, flies that survive through this initial stage can ultimately live longer than control flies (more than 80 days). It may depend on individual differences in ATP and adenosine levels.

On the contrary, flies with lower dAdoR level, although protected against early death, later showed higher mortality than control flies. Their survival decreased quite abruptly, probably because of the lack of adenosine signaling. Even in 30-day-old flies we observed higher mRNA *dAdoR* level than in 7-day-old. This leads to the conclusion that in 30-day-old flies the level of dAdoR and/or adenosine already decreases. These results imply that a proper physiological level of adenosine and its receptors, is important for survival while chronic overproduction of adenosine leads to pathological conditions (Borea et al. 2017).

It is not surprising that changes in adenosine signaling also affect fitness, examined in our study using the climbing assay. This assay is a simple method that enables quick and easy screening of climbing deficits in flies, thus providing information on their physiological aging (Jones and Grotewiel 2011).

We found that adenosine level is a detrimental factor in maintaining fitness. In humans, the role of adenosine in maintaining fitness has also been reported (Simpson et al. 1992; Purpura et al. 2017). Athletes involved in various sports require interval training to achieve maximum performance, and this training induces higher levels of adenosine production due to the increased rate of utilization of ATP. In our studies on fitness, the experimental group of 60-day-old flies showed a drastic decline in fitness with the silenced *dAdoR* gene. This effect was reversed in older- and middle-aged females and older males after increasing the abundance of dAdoR in all neurons. The higher level of dAdoR in glial cells, after overexpression of *dAdoR* in all glia, was also beneficial for the fitness of the flies. These results suggest that adenosine influences the adaptive responses necessary for improved performance (Simson and Phillis 1992). Oral administration of adenosine to athletes improved their strength, lean body mass, blood flow, and increased power and performance (Purpura et al. 2017). We observed a similar effect after enhancing adenosine signaling in older flies, in which the physiological level of dAdoR in neurons and glial cells declines with age. So, changes in adenosine and its receptor levels are observed when they occur in the whole nervous system, but when they are restricted to some neurons as photoreceptors (Lankford et al. 2020), changes in fitness were not observed. But in survival and sleep, as well as in cellular studies, we found significant changes. They can be attributed to the fact that *GMR*-*Gal4* (a driver in the eye photoreceptors) shows a broad expression profile. *GMR* is also present in neurons in the ventral ganglia in the second and third instar larvae (Freeman 1996; Ray and Lakhotia 2015).

Adenosine is a popular and well-known somnogenic agent that increases sleep in mammals (Porkka-Heiskanen et al. 1997, 2002). The A2AR receptors are excitatory and are known to promote sleep. Adenosine A2A receptor agonists, administered to the subarachnoid space adjacent to the basal forebrain area, help induce sleep (Satoh 1999).

Sleep in the fruit fly and mammals has similarities that appear to be evolutionarily conserved. One of the hypotheses about sleep function is that it is time for the remodeling of the synapse in the brain (Tononi and Cirelli 2006). Like the application of adenosine, the A1 receptor agonist cyclohexyl adenosine was found to increase sleep in both flies and mammals (Hendricks et al. 2000). Our results showed that an excess of dAdoR induces more sleep at night and increases total sleep of flies, just as in rodents, while suppressing the expression of the *dAdoR* gene does not affect sleep. *dAdoR* overexpression also affects *siesta*, which was longer than in the control.

Adenosine has many physiological functions throughout the nervous system (Latini and Pedata 2001). As a neuromodulator, it plays a role in fine-tuning of synaptic transmission (Phillis and Wu 1981). Although both A1 and A2 receptors are involved in such actions, it seems that A2AR is a key player in the regulation of neuromuscular transmission (Nascimento et al. 2014).

Analyses of the presynaptic protein BRP level in the lamina tetrad synapses, between the photoreceptor terminals and postsynaptic cells, and its daily plasticity were affected after silencing of *dAdoR* in photoreceptors or in glial cells. Since BRP is a presynaptic scaffolding protein of synapses in the central and peripheral nervous systems, changes in the level of BRP after silencing *dAdoR* in photoreceptors and glial cells suggest an involvement of adenosine and dAdoR in neurotransmission in time-dependent manner.

BRP after silencing *dAdoR* in photoreceptors still maintains its daily oscillations, but this daily pattern is disrupted after silencing *dAdoR* in glial cells. Glial cells are known as circadian oscillators that affect circadian rhythms in various processes, including the rhythm in activity and sleep (Damulewicz et al. 2022). It indicates that lower expression of BRP after silencing *dAdoR* may have an impact on fitness. Previous studies have shown that lower expression of BRP affects the ultrastructure of the synaptic active zone (AZ) in neuromuscular junctions in *Drosophila* (Wagh 2006). Flies with lower expression of BRP could not sustain stable flight and crashed to the ground (hence, Bruchpilot) (Wagh 2006).

A similar effect was observed in the fitness study. BRP maintains the structure of AZ and proper neurotransmission (Wagh 2006). In control flies, the BRP level in tetrad synapses increased during the first part of the day and at the beginning of the night. The first peak was attributed to the blue-light-sensitive protein CRY (cryptochrome) that is responsible for the degradation of BRP, resulting in its higher synthesis (Damulewicz et al. 2020). The second peak is generated by the circadian clock. Two peaks, first during the beginning of the day (ZT1) and second during the beginning of the night (ZT13) can be observed only in light/dark conditions, but in constant darkness only the second peak is maintained (Górska-Andrzejak et al. 2013).

These changes in BRP level correspond to the rhythm in locomotor activity and in the number of tetrad synapses in the lamina photoreceptor terminals (R1–R6) (Woźnicka et al. 2015), as well as in the size and shape changes of their postsynaptic partners L1 and L2 large monopolar cells (LMC) (Pyza and Meinertzhagen 1995). These LMCs in *Drosophila* swell twice during the day, at the beginning of the day and at the beginning of night (Pyza and Meinertzhagen 1999).

In our study, we focus on the lamina because it provides a convenient part of the brain for studying various processes in the central nervous system, such as synaptic plasticity. Our observations from longitudinal sections of the lamina cartridges showed that the level of the BRP protein changes during the day in the depth of the distal and proximal parts of the lamina. The level of this protein in the distal lamina was comparatively higher than in the proximal lamina in both the experimental and control groups. Although we observed cyclical changes in BRP level at other time points, at ZT13, we found that the level of this protein was high in both experimental and control groups during high locomotor activity of flies.

Furthermore, we observed the lowest expression of BRP in the lamina of the experimental group at the beginning of the day (ZT1) when *dAdoR* was silenced in glia cells. This resulted in the lack of the morning peak of BRP. The absence of the morning peak indicates the involvement of glial cells via dAdoR in synaptic transmission in tetrad synapses during the day. While at the beginning of the night, the BRP level is lower than in the controls, but it is still higher than that observed at the beginning of the day. In the later part of the night, the level of BRP in both the distal and proximal lamina did not show significant changes between the experimental and control groups.

The future challenge will be to decode how adenosine signaling works. The highlight of this study is that overexpression of dAdoR is a potent factor in promoting sleep. Furthermore, adenosine plays an important role in neurons and glial cells affecting synaptic plasticity and, as a result, survival and fitness of flies. We also believe that this basic study on adenosine receptors creates a framework for future studies in invertebrates where we encounter a huge knowledge gap, unlike their vertebrate counterpart.

## Conclusions

In our study, we found that overexpression of *dAdoR* promotes sleep during the night and decreases activity, just as in vertebrates. The study provided a new and different perspective on the role of dAdoR in the promotion of fitness and survival based on tissue specificity and showed the role of dAdoR in the middle-age survival of fruit flies. We conclude that aging influences the expression of dAdoR in neurons that delays the senescence of negative geotaxis in middle-age flies with progression in old age. In addition to this, the invertebrate AdoR is a key player in fine-tuning communication between neurons and glia. Adenosine signaling decreases when flies are old. This is an effect in the decrease in ATP and adenosine production with age. The effect of dAdoR may be strain-specific and tissue-specific, but our study showed important functions of adenosine receptors in the regulation of sleep, longevity, fitness, and synaptic plasticity.

## Supplementary Information

Below is the link to the electronic supplementary material.

Supplementary file1 (JPG 883 KB)

Supplementary file2 (DOCX 16 KB)

## Data Availability

The raw data supporting the conclusions of this article will be made available by the authors, without undue reservation. Data will be also available from the Jagiellonian University data repository RODBUK.
