# Supplementary figures and images for: Effects of adenosine receptor overexpression and silencing in neurons and glial cells on lifespan, fitness, and sleep of Drosophila melanogaster
Source: Exp Brain Res. 2023 Jun 19;241(7):1887–904. doi: 10.1007/s00221-023-06649-y (PMC10348948; doi:10.1007/s00221-023-06649-y)

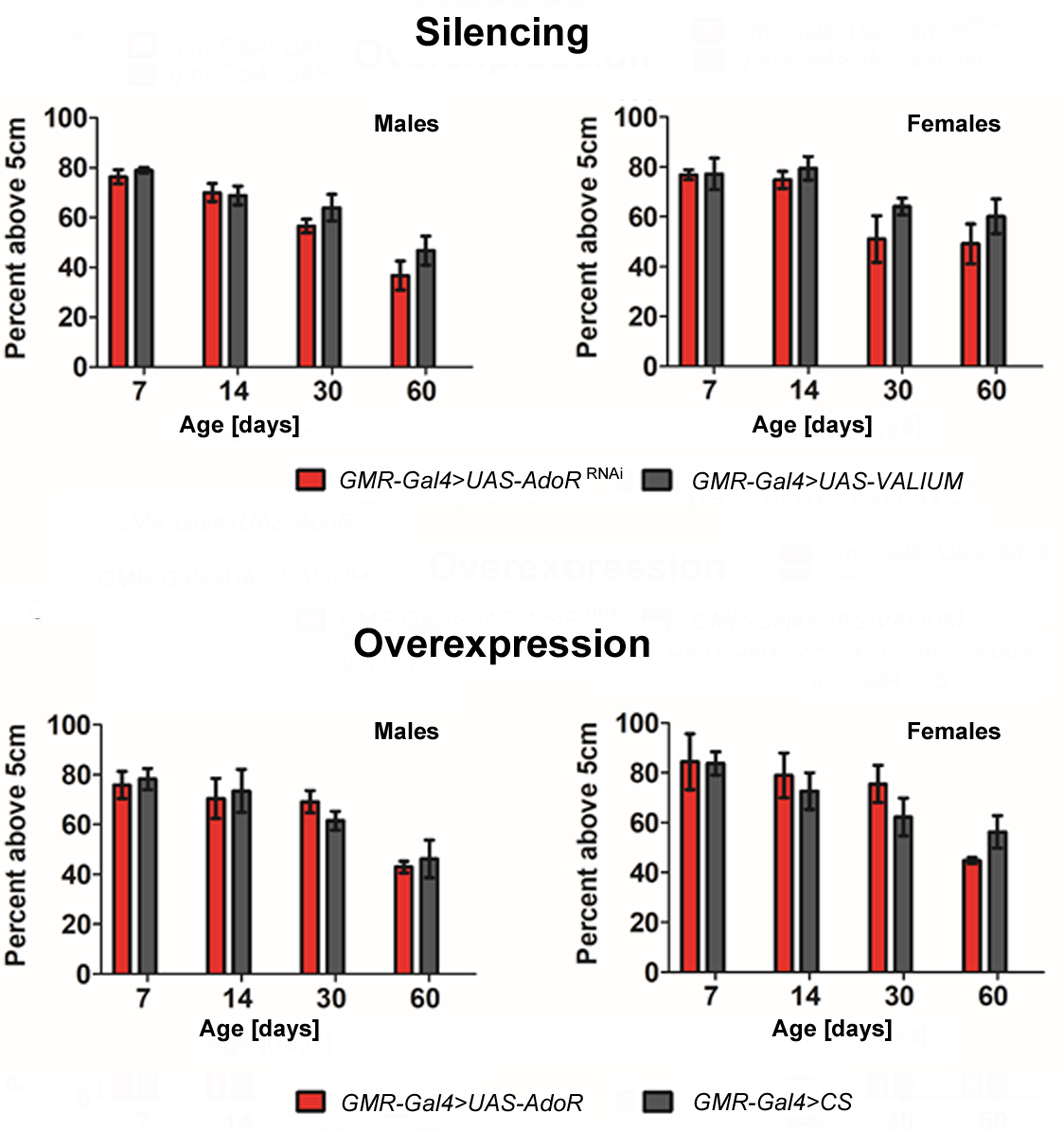

Supplement: Supplementary file 1 — Supplementary file1 (JPG 883 KB) [file 221_2023_6649_MOESM1_ESM.jpg]
