# Supplementary material for: Effects of adenosine receptor overexpression and silencing in neurons and glial cells on lifespan, fitness, and sleep of Drosophila melanogaster
Source: Exp Brain Res. 2023 Jun 19;241(7):1887–904. doi: 10.1007/s00221-023-06649-y (PMC10348948; doi:10.1007/s00221-023-06649-y)
Supplement: Supplementary file 2 — Supplementary file2 (DOCX 16 KB) [file 221_2023_6649_MOESM2_ESM.docx]

**Table 1** Results of the Kruskal–Wallis test (K-W test) and the *post-hock* *Conover-Iman’s* test (C-I test; p<0.05 in all cases) for the climbing assay of 7-, 14-, 30- and 60-day-old males (grey lines) and females (white lines) with *AdoR* overexpression and silencing in photoreceptor cells, as well as their controls.

| Groups | K-W test  Statistic | K-W test  *p* value | C-I test | |
| --- | --- | --- | --- | --- |
| *GMR*-*Gal4* >*UAS*-*AdoR* | 7.2 | ns | ns | |
| *GMR*-*Gal4* >*CS* | 8.8 | <0.05 | 7, 14 vs 60 | |
| *GMR*-*Gal4* >*UAS*-*AdoR* | 7.3 | ns | ns | |
| *GMR*-*Gal4* >*CS* | 8.6 | <0.05 | 7, 14 vs 60 | |
| *GMR*-*Gal4* > *UAS*-*AdoR* ^RNAi^ | 10.1 | <0.01 | 7 vs 30, 60; 14 vs 60 | |
| *GMR*-*Gal4* >*UAS-VALIUM* | 9.6 | <0.05 | 7 vs 30, 60; 14 vs 60 | |
| *GMR*-*Gal4* > *UAS*-*AdoR* ^RNAi^ | 8.5 | <0.05 | 7 vs 30, 60; 14 vs 60 | |
| *GMR*-*Gal4* >*UAS-VALIUM* | 8.7 | <0.05 | 7 vs 60; 14 vs 30, 60 | |
| MALES |  |  |  |  |
| FEMALES |  |  |  |  |
